# A visual review of the interactome of LRRK2: Using deep-curated molecular interaction data to represent biology

**DOI:** 10.1002/pmic.201400390

**Published:** 2015-03-21

**Authors:** Pablo Porras, Margaret Duesbury, Antonio Fabregat, Marius Ueffing, Sandra Orchard, Christian Johannes Gloeckner, Henning Hermjakob

**Affiliations:** 1 European Molecular Biology Laboratory, European Bioinformatics Institute (EMBL-EBI)Hinxton, UK; 2 Research Unit Protein Science, Helmholtz Zentrum München – German Research Center for Environmental HealthNeuherberg, Germany; 3 Medical Proteome Center, Institute for Ophthalmic Research, Eberhard Karls University TübingenTübingen, Germany; 4 German Center for Neurodegenerative Diseases (DZNE)Tübingen, Germany

**Keywords:** Bioinformatics, Curation, Data visualization, Molecular interaction database, Parkinson's disease, Protein interaction network

## Abstract

Molecular interaction databases are essential resources that enable access to a wealth of information on associations between proteins and other biomolecules. Network graphs generated from these data provide an understanding of the relationships between different proteins in the cell, and network analysis has become a widespread tool supporting –omics analysis. Meaningfully representing this information remains far from trivial and different databases strive to provide users with detailed records capturing the experimental details behind each piece of interaction evidence. A targeted curation approach is necessary to transfer published data generated by primarily low-throughput techniques into interaction databases. In this review we present an example highlighting the value of both targeted curation and the subsequent effective visualization of detailed features of manually curated interaction information. We have curated interactions involving LRRK2, a protein of largely unknown function linked to familial forms of Parkinson's disease, and hosted the data in the IntAct database. This LRRK2-specific dataset was then used to produce different visualization examples highlighting different aspects of the data: the level of confidence in the interaction based on orthogonal evidence, those interactions found under close-to-native conditions, and the enzyme–substrate relationships in different in vitro enzymatic assays. Finally, pathway annotation taken from the Reactome database was overlaid on top of interaction networks to bring biological functional context to interaction maps.

## 1. Introduction

As proteins form larger assemblies to confer context-specific functionality 1, the systematic analysis of protein–protein interactions (PPIs) is a powerful strategy for identifying physiological pathways associated with a protein of interest, following the “guilt of association” principle, and to further characterize these pathways by unveiling the physical contacts that underlie them 2. However, when using experimentally derived molecular interaction information stored in databases, the researcher must address multiple challenges, including those of data quality, the degree of coverage of their interactome of choice and the heterogeneity in the representation of interactions 3,4. Since the quality and reliability of the data largely depend on the experimental approaches used to detect the interactions, great effort is made to represent this information accurately and make it available to the researcher. Biocurators working for primary databases 5 such as IntAct 6, Molecular INTeraction database (MINT) 7, MatrixDB 8, Database of Interacting Proteins (DIP) 9, or BioGRID 10 extract interaction evidence from published literature following defined rules, but these rules are not always defined by a community and can vary from database to database. Secondary resources aim to offer a more comprehensive view of the interactome by either merging several of these primary, curated datasets (Agile Protein Interaction DataAnalyzer (APID) 11, mentha 12, Human Integrated Protein-Protein Interaction rEference (HIPPIE) 13) or by adding computationally predicted unexplored interactions based on the experimental data (Search Tool for the Retrieval of Interacting Genes/Proteins (STRING) 14, Unified Human Interactome (UniHI) 15). However, the need to reconcile different representation strategies often results in the sacrifice of detailed information unique to specific databases.

Data integration became easier with the creation of the Human Proteome Organisation Proteomics Standards Initiative for Molecular Interactions (HUPO PSI-MI) 16, an international initiative that resulted in the creation of the PSI-MI XML format for the representation of interaction data and the minimum information required for reporting a molecular interaction experiment (MIMIx) 17. The International Molecular Exchange consortium (IMEx), an international collaboration between major interaction data providers, further developed the standards and helps coordinating and sharing curation efforts, following the same set of guidelines and supporting community-defined standards (www.imexconsortium.org) to produce a nonredundant dataset 18. The detailed IMEx curation model means that the data can subsequently be subjected to sophisticated filtering and analysis, for example searching experimental evidence according to the host system in which they were generated, producing a tissue-specific interactome. Meta-databases such as STRING and mentha or some services accessing the data through the Proteomics Standard Initiative Common Query Interface (PSICQUIC) protocol 19 still lose part of this detailed information in order to reconcile IMEx entries with data from providers that do not curate to this level. Nevertheless, the work of the IMEx consortium has resulted in an overall improvement in data collection standards.

One of the main challenges researchers face when using molecular interaction information is how to meaningfully visualize the full extent of the available data. Many questions can be addressed by achieving a useful representation; however there is no “correct” visualization strategy. A successful approach for a small subset of interactions will fail when applied to a large interaction dataset and vice versa. Integrating external information such as annotations or –omics data (e.g. fold changes in mRNA/protein transcript levels following cellular stimulation) adds further complexity. The many tools and approaches to visualize and analyze interaction networks have been reviewed elsewhere 20,21.

Here, we present a detailed visual analysis strategy for functional protein networks, using the example of LRRK2, a protein mutated in Parkinson's disease (PD). PD is the second most common age-related neurodegenerative disease and is clinically characterized by movement impairments, bradykinesia, rigidity, and resting tremor and pathologically by the progressive loss of dopaminergic neurons in the *substantia nigra* and the formation of Lewy bodies 22. Although the majority of cases are sporadic, familial forms of PD exist. Among these cases, mutations in the LRRK2 gene (PARK8; Online Mendelian Inheritance in Man (OMIM) 609007) have been found in 5–15% of families with late-onset autosomal dominant PD 23–25. LRRK2 belongs to the Roco protein family of GTPases, with a Roc (Ras of complex proteins) GTPase domain, a COR dimerization region, and a protein kinase domain. PD-associated mutations within the enzymatically active domains lead to reduced GTPase and increased kinase activities 26–29. LRRK2 also contains four predicted repeat structures: the N-terminal ankyrin, armadillo, the leucine-rich repeat regions, and a C-terminal WD40 domain, all of which have been associated, by similarity, with protein interactions. Recent work demonstrates a function of LRRK2 in cytoskeletal dynamics, vesicular transport, including the transport of synaptic vesicles, and autophagy. Pathogenic mutations of LRRK2 have been found to alter these processes, suggesting that the neuronal death associated with PD is caused by their perturbation (reviewed in 30–32). In addition, LRRK2 has been associated with the innate immune system and neuroinflammation (reviewed in 33,34).

**Figure 1:**
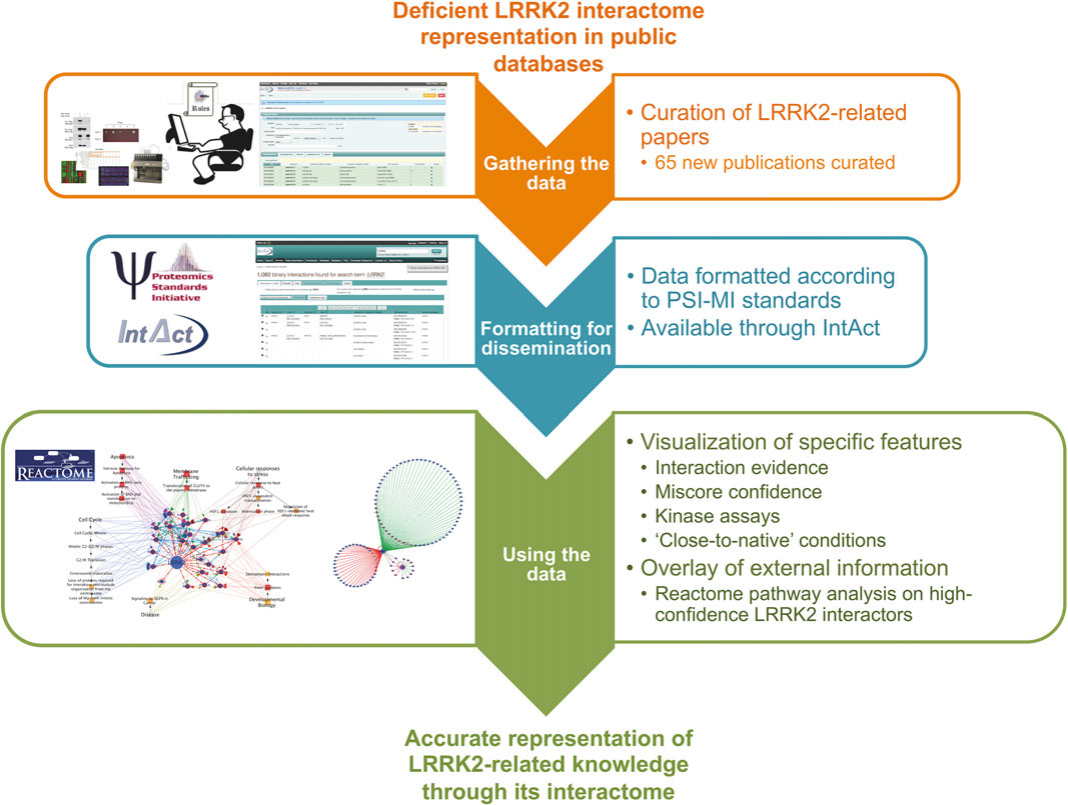
Graphical abstract. After detecting the poor coverage for LRRK2-related interactions, the IntAct team performed targeted curation of the experimental evidence available in the literature. This dataset has been made available through IntAct using international standards and several visualizations highlighting different features in the dataset are presented in this work.

With LRRK2 becoming a major player in Mendelian forms of PD, several studies have been conducted to systematically identify LRRK2-associated protein complexes in order to understand its physiological function and the molecular pathophysiology of LRRK2 mutations 35–38. We have curated published interaction data and visualized the experimental interaction data with the aim of supporting experimental groups in their effort to discover novel pathways relevant to the disease. Figure 1 provides a workflow in which the main procedures followed to generate the data described in this publication are highlighted.

## 2. Curating the LRRK2 interactome

### 2. 1. IntAct coverage of the LRRK2 interactome

Prior to the start of the targeted curation project in April 2012, IntAct contained only four articles featuring 69 binary interactions involving the human or mouse LRRK2 protein. Each distinct piece of evidence for a given pair of interacting molecules is considered a separated binary interaction in this data schema, which means that these 69 binary interactions featured 55 interacting protein pairs. By October 2014, 61 new publications featuring 1006 interactions had been added, resulting in a total of 1075 binary interactions for 612 interacting pairs and 598 molecules, predominantly proteins (Supporting Information Table 1). Interactions are reported in high detail and available through the PSI-MITAB 2.7 format, a human-readable, tab-delimited simple format that can be managed and opened in any text editor or in Excel (see https://code.google.com/p/psicquic/wiki/MITAB27Format). The LRRK2 interactome dataset includes the majority of relevant new publications published in this period, primarily captured by IntAct curators at the EBI but also by many of the other resources that use IntAct as a curation platform 6 (Supporting Information Table 2). In order to subsequently access this information, interaction data for human and mouse LRRK2 were queried via the IntAct website (www.ebi.ac.uk/intact) on October 7, 2014 (IntAct release 183), using the UniProtKB accessions for both proteins in the query “Q5S007 OR Q5S006.” Query results in PSI-MITAB 2.7 can be found in the Supporting Information file lrrk2_mitab27.txt. Up-to-date interaction data for human and mouse LRRK2 can be accessed via this link: http://www.ebi.ac.uk/intact/pages/interactions/interactions.xhtml?query = Q5S007%20OR%20Q5S006.

### 2. 2. LRRK2 interactions outside IntAct

In order to assess the coverage of LRRK2 interactome in other interaction resources associated with the IMEx consortium, we used PSICQUIC View (www.ebi.ac.uk/Tools/webservices/psicquic/view) to query the DIP 9 and BioGRID 10. While no additional interactions were found in DIP, 305 interactions were found in BioGRID. However, this database has not yet adopted the IMEx curation guidelines, and its data cannot be completely integrated with IntAct data. Supporting Information Table 3 shows that 31 publications were curated in BioGRID and not in IntAct, giving a total of 95 interaction pieces of evidence present in BioGRID but excluded from further analysis.

Finally, we queried other non-IMEx compliant resources including the Human Protein Reference Database (HPRD, www.hprd.org 39) and CCSB Interactome Database (http://interactome.dfci.harvard.edu/H_sapiens/ 40) through their respective websites. HPRD is a centralized platform focused in human data regarding disease association, PTMs, domain architecture, and others, plus a sizeable amount of protein interactions. The CSSB Interactome Database is a portal that provides access to a large dataset produced by Marc Vidal's lab using their high-throughput yeast two-hybrid technology. Unfortunately, no interactions involving mouse or human LRRK2 are found in either of these resources. HPRD ceased active curation in 2010, but the CSSB Interactome Database is a project under development and new interactions are regularly added, so it may add LRRK2 interactions in the future.

## 3. Visualizing the LRRK2 interactome

### 3. 1. The MITAB tabular format

As described above, we downloaded LRRK2-related interactions from the IntAct website, choosing MITAB 2.7 as our format of choice. MITAB is a derivation from the PSI-MI XML format adapted to provide a simple, tabular format 41. Details about the 42 fields provided in its 2.7 version can be found in the PSI-MI Google code page (https://code.google.com/p/psimi/wiki/PsimiTab27Format). The format can be opened in any text editor or Excel and can be easily parsed due to the simplicity of its structure. In this format every line represents a piece of evidence for interaction between two molecules. Interactions in which more than two molecules are involved, and for which the binary relationships are not known (e.g. protein complexes pulled down through affinity chromatography), are converted by IntAct to binary pairs using the spoke expansion algorithm 42. The columns in the format represent different fields providing information such as identifiers for the participants in the interaction, the method used to provide the evidence reported, the publication in which it was found, or whether the participant was full length or just a fragment of a protein.

In order to make effective use of the downloaded file, it is often useful to perform basic parsing of the format. We chose to simplify the fields for the participant and publication identifiers and added three more columns to help exploring the features annotated for the participants: lrrk2_construct, lrrk2_mutation, lrrk2_fragment. The “features” fields contain specific details about the molecules taking part in each particular interaction experiment, such as whether they have tags attached or if a fragment of the protein was used rather than the full-length molecule. The newly created fields hold Boolean values of “yes” and “no” if the “Feature(s) interactor A/B” field meets certain conditions:

- lrrk2_construct: equals “yes” if the field contains tags or delimited regions.
- lrrk2_mutation: equals “yes” if the field contains mutation information (whether it affects binding of the interacting partners or not).
- lrrk2_fragment: equals “yes” if field contains delimited regions.

An additional file (Supporting Information file nodecolumns_lrrk2.txt) was generated to provide basic information about the participants derived from the MITAB file: molecule name, type, species, and identifier for mapping purposes. The network representation and analysis tool Cytoscape 43 (version 3.2) was used to import these processed files and produce suitable representations. A Cytoscape session file holding these visualizations can be found in the Supporting Information data (lrrk2_networks.cys). Since the figures we provide contain a great deal of detail, they are difficult to examine as static images. The Cytoscape session file allows for more convenient navigation, allowing the user to zoom into regions of interest and highlight specific aspects of the network. Additionally, we created web browser accessible visualizations via the CyNetShare tool (http://idekerlab.github.io/cy-net-share/). This browser-based web application renders JSON-formatted network visualizations created via Cytoscape 3.2. These visualizations are accessible using any web browser and allow zooming into the network and moving nodes. Links are given in the corresponding figure legends.

### 3. 2. The LRRK2 interactome by interaction detection method

Every experimental PPI detection method generates false positives and negatives, and thus the resulting interactome maps are both noisy and incomplete. There have been several efforts to compare different experimental methodologies and provide unbiased estimations of their accuracy 44–48; however it has become apparent that each technique samples different sections of the interactome and can add value to the overall picture. Thus, a common approach to assessing the validity of interactions is to combine different experimental strategies in an orthogonal fashion. Since interactomes derived from public databases such as IntAct feature interactions obtained through multiple approaches, we focused our first visualization approach on highlighting the interaction detection method.

**Figure 2:**
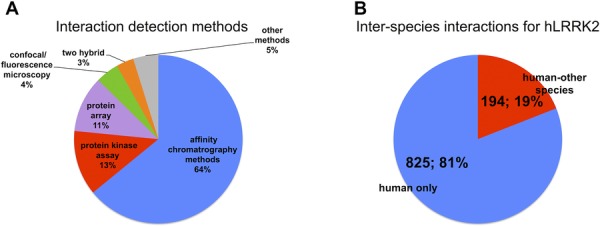
Interactions involving LRRK2/Lrrk2 in the IntAct database (I). (A) Proportion of different experimental methodologies used to demonstrate interactions for human LRRK2 and mouse Lrrk2, described using the PSI-MI controlled vocabulary. Percentage values are shown for each category. An extended version of this figure can be found in Supporting Information Fig. 1. (B) Interspecies interactions for human LRRK2 as reported in IntAct. “Human-only” interaction pieces of evidence are those in which only proteins of human origin were used. “Human-other species” accounts for those in which LRRK2 was tested against proteins from another organism. Total number of interactions and percentage is shown for each category.

**Figure 3:**
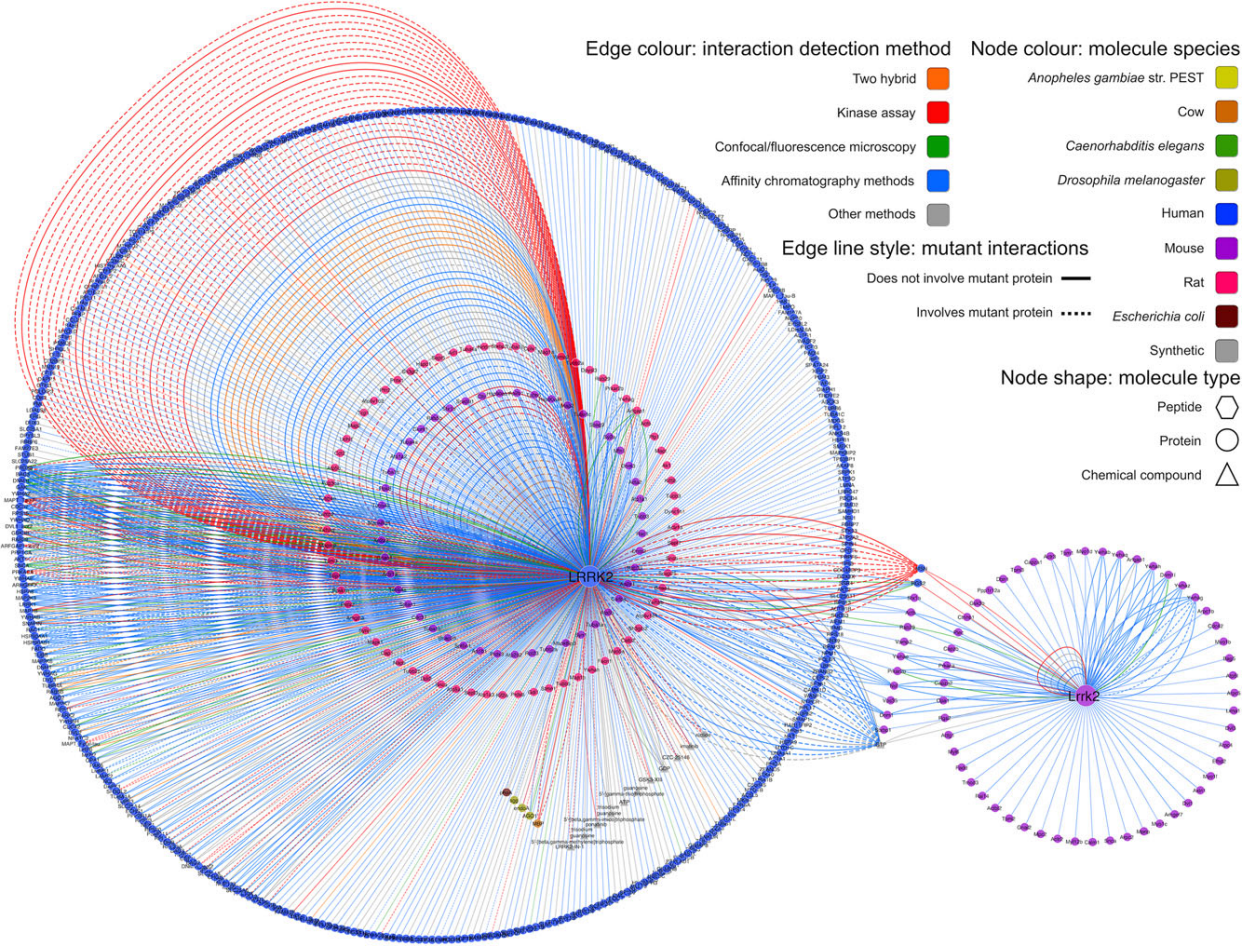
Interactions involving LRRK2/Lrrk2 in the IntAct database (II). An interaction network depicting all interactions found for LRRK2/Lrrk2 in IntAct, with the node color reflecting the protein species and the edge color reflecting the interaction detection method (see legend). Each interaction piece of evidence is represented as a single edge and interactions involving mutant forms of the proteins are depicted as dashed lines. A web visualization of this network is available at http://tinyurl.com/lrrk2-f3.

Six hundred eighty-nine interactions of the 1075 LRRK2 interactions downloaded from IntAct have been found using affinity chromatography based methods, such as pull-down or coimmunoprecipitation (see Fig. 2A and Supporting Information Fig. 1). A significant portion of the interaction evidence (134 interactions) originates from in vitro kinase assays. Other methods used include protein arrays, two-hybrid assays, surface plasmon resonance, and fluorescence polarization spectroscopy. In Fig. 3 detection methods were mapped to the edge color and each edge represents single interaction evidence (i.e. an interaction detected in a publication, using a specific method in a particular host organism). By using this “redundant edges” visualization, it is easy to recognize which molecule pairs have been found in different publications, since they are connected by multiple lines, and to deduce if they have been orthogonally validated, using the edge color as a guide to the interaction detection method. It is immediately obvious that a significant proportion of the data relates to the human LRRK2 protein self-interacting, while autophosphorylating.

It is important to emphasize that, in all our representations, nodes/proteins were colored according to the organism of origin. The node color allows easy identification of interspecies interactions (see Fig. 2B), often explained by the use of close orthologs as experimental substitutes for the protein from the species of interest. Node shape also indicates if the molecule represented is a protein, chemical compound, or synthetic peptide. Although they can also be represented in IntAct, no interactions with genes or nucleic acids were present in our LRRK2/Lrrk2 dataset.

### 3. 3. Detailed features I: “Close-to-native” interactions

**Figure 4:**
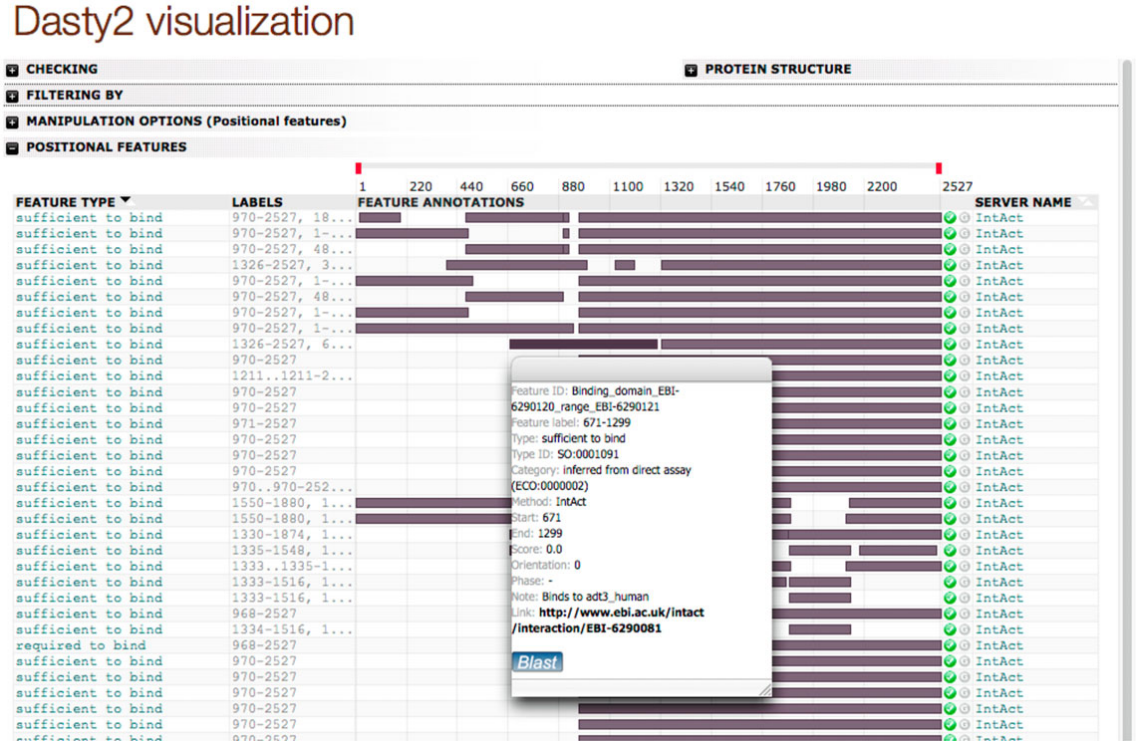
Human LRRK2 and mouse Lrrk2 construct information. An overview of the different fragments that have been used to test LRRK2 interactions as shown by the Dasty2 visualization featured in the IntAct website (see http://www.ebi.ac.uk/intact/molecule/EBI-5323863 for a full version). Different LRRK2 fragments found to bind in different interaction experiments are represented as dark-gray boxes mapped against a ruler of full-length LRRK2.

IntAct's high-detail, curated information can be visualized using other complementary strategies that can help identifying bona fide interacting partners of LRRK2. The researcher might want to consider only methods that detect interactions under in vivo, “close-to-native” conditions, avoiding strategies that require the use of heterologous expression systems or information derived from the use of protein fragments. We have applied such strategy to our LRRK2 dataset using the additional fields created while parsing the MITAB 2.7 result file. Due to the challenges posed by the size of the LRRK2 protein to in vitro experimentation, the majority of the molecular interaction evidence found for human LRRK2 was obtained using fragments of the protein expressed in heterologous systems (94% tested in heterologous vs. 6% in homologous systems). Most of these fragment-derived interactions involved the WD40 repeat containing domain (approx. residues 2124–2527), the ROC GTPase domain (approx. residues 1328 to 1513), or a bigger fragment spanning the whole C-terminal region (approx. residues 970 to 2527) to include the leucine-rich repeats, ROC GTPase domain, kinase domain, and WD40 domain. IntAct's detailed curation model allows for a representation of the binding regions mapped in IntAct for human LRRK2, as shown in the screenshot displayed in Fig. 4. This view uses Dasty2 49 to display different features associated with the LRRK2 sequence, such as mapped domains taken from UniProt or peptides detected in MS experiments as stored in the PRIDE database. In this case, we are displaying fragments used in interaction experiments as annotated in IntAct. The full representation can be accessed at www.ebi.ac.uk/intact/molecule/EBI-5323863 and hovering with the mouse on top of the fragments displays the range details and other specific information. Mouse Lrrk2 interactions, on the other hand, seem to have been largely tested using the full-length molecule (only two experiments using fragments recorded in IntAct), although heterologous expression systems and tagged constructs have been widely used. Supporting Information Fig. 2 displays the experimental organism in which the interaction was demonstrated as the edge color. Of the 1075 human or mouse interactions, only a small fraction have been observed using untagged, full-length protein tested in native tissue. Figure 6A represents the following: 34 interactions involving 23 proteins for mouse (left-hand figure) and only 10 involving 5 proteins for human (right-hand figure). Detection methods reported are coimmunoprecipitation, colocalization, SDS-gel comigration, and density gradient cosedimentation. Such “close-to-native” methodologies do not provide evidence for a binary relationship but they are complementary and add confidence to those interactions obtained using fragment constructs, heterologous expression systems, and in vitro methods.

### 3. 4. Detailed features II: MIscore

**Figure 5:**
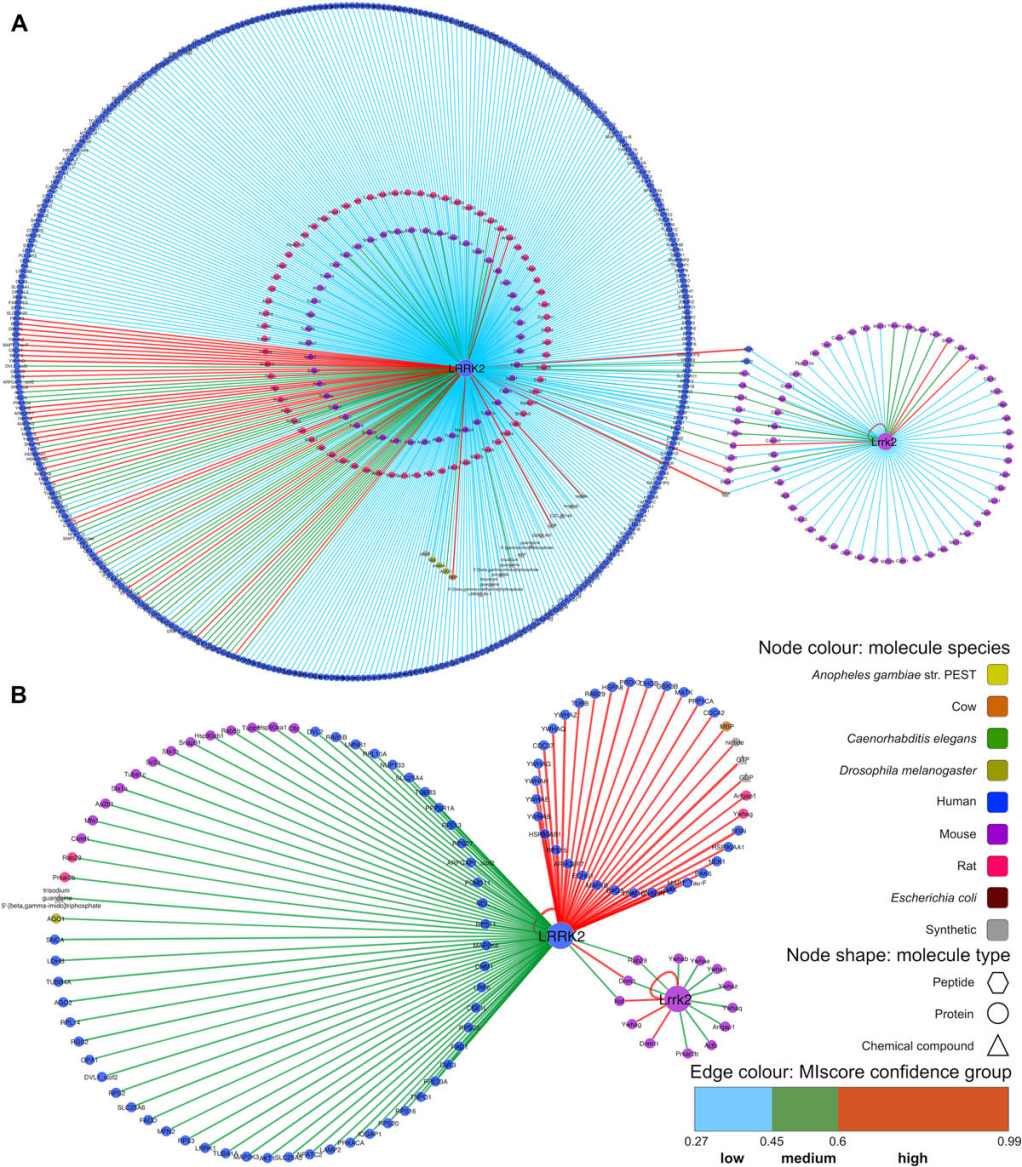
Visualization of confidence based on experimental evidence in human LRRK2 and mouse Lrrk2 networks. (A) Full LRRK2/Lrrk2 interaction network where multiple pieces of evidence have been collapsed into single edges and MIscore values for each edge mapped to the edge color. A web visualization of this network is available at http://tinyurl.com/lrrk2-f5a. (B) Medium- and high-confidence interval interactions for LRRK2/Lrrk2. A web visualization of this network is available at http://tinyurl.com/lrrk2-f5b.

**Figure 6:**
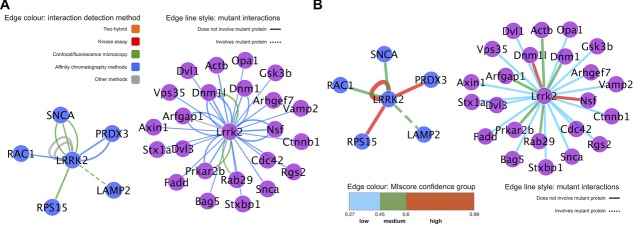
Interactions for LRRK2/Lrrk2 tested under “close-to-native” conditions. (A) As in Fig. 2, node color is mapped to protein species, edge color to interaction detection method and dashed edges indicate interactions involving or affected by mutant forms of the participants. A web visualization of this network is available at http://tinyurl.com/lrrk2-f6a. (B) Interactions for LRRK2/Lrrk2 found under “close-to-native” conditions, with collapsed edges and MIscore represented as edge color. See network legend for nodes in (A) and (B) in Fig. 2. A web visualization of this network is available at http://tinyurl.com/lrrk2-f6b.

There is no generally accepted method for establishing the reliability of the interaction between a pair of proteins 19, but confidence scoring systems exist that make use of reiterative evidence, detection methods, and/or parameters such as coexpression values or the topology of the network itself 13,14,50,51. The scoring method MIscore (Molecular Interactions score) is already in use in IntAct and PSICQUICView and weights the annotation evidence available in the PSI-MI output formats 89 to give a value to the accumulated evidence supporting an interacting pair of molecules, which can be viewed as a measure of reliability (https://code.google.com/p/miscore/). This can then be used to select only well-characterized, orthogonally validated interactions and allows a simpler representation of fewer, high-confidence interactors. However, this scoring system penalizes less-characterized interactions. For this publication, we define three classes of confidence using MIscore: high confidence, with an MIscore value equal or greater than 0.6, medium confidence, with an MIscore cutoff ≥ 0.45 and < 0.6; and low confidence if the MIscore values are under 0.45. LRRK2 interactors with an MIscore value under 0.45 have been identified exclusively in a single publication, using only one specific methodology. Over this threshold, interactions have been confirmed orthogonally and become more reliable. These three regions of confidence were visualized as edge colors in a simplified network for LRRK2, in which the multiple edges derived from different evidence types have been collapsed into a single edge connecting interacting molecules (Fig. 5A). For clarity, the subset of interactions that score in the medium- and high-confidence intervals can be represented in an additional network and separated in the display (Fig. 5B). We also applied our MIscore-based visual style over the subnetwork of interactions found under “close-to-native” conditions (Fig. 6B), highlighting those interacting partners found under these conditions and confirmed orthogonally through other methods.

### 3. 5. Detailed features III: Kinase assays

**Figure 7:**
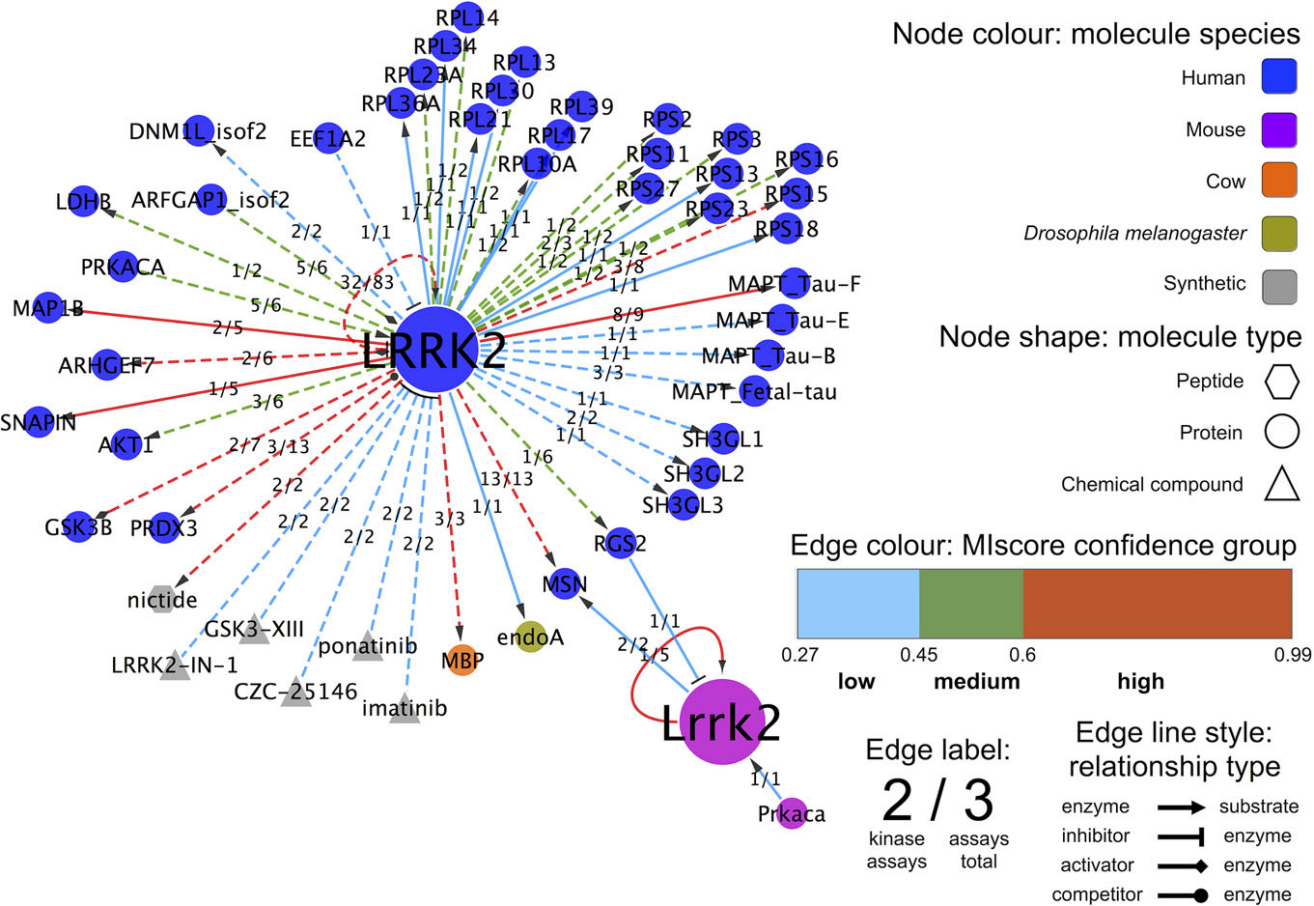
LRRK2 in vitro kinase assays: Simplified interaction network in which only those molecules involved in kinase assays have been represented. Edges have been collapsed so every pair of molecules is connected by just one edge and colored by MIscore confidence group. The number of underlying edges based in kinase assays by the total number of evidences for a given pair is shown as a fraction on top of every edge. The enzyme–substrate relation is visualized using arrows, substrates being the arrow targets. Other biological roles such as “inhibitor” or “stimulator” of enzymatic activity are also represented with appropriate symbols. For the remaining visual features, see legend. A web visualization of this network is available at http://tinyurl.com/lrrk2-f7.

LRRK2 is a protein kinase and a mutation in its kinase domain has been linked to familial forms of PD 24–26,28. The IMEx curation model records enzymatic assays as acceptable interaction evidence, but only when they are performed in vitro by directly interacting purified proteins 18. We generated a specific display in which a small network of enzymatic assay-derived LRRK2 interactions is represented. Evidence for three different classes of in vitro enzyme assays have been curated for LRRK2 and visualized as different edge color in Supporting Information Fig. 3: GTPase and kinase assays in which LRRK2 acts mainly as an enzyme, and assays in which LRRK2 is a substrate. Focusing on the kinase assays, which account for most of the evidence, different groups of in vitro substrates and molecules that influence LRRK2 kinase activity were grouped for ease of visualization. We created a simplified view in which all interaction evidence are collapsed into a single edge and our global MIscore is represented as the edge color (Fig. 7). Edges were labeled with the ratio of the number of kinase assay evidence to the total number of evidence for each interacting pair. This allows easy identification of those potential LRRK2 kinase substrates that have been either repeatedly tested in different publications or supported by additional interaction evidences.

### 3. 6. Interpreting LRRK2 interaction networks

A confidence scoring system, such as MIscore, could be the most direct way to produce a draft list of putative bona fide LRRK2 interacting partners. However, caution must be taken when considering the limitations of any score. MIscore is dependent on the number of reports for a given interaction, especially when evidence is given in separate publications. A high MIscore value is generally indicative of interactions for which abundant, good quality data are available. However, commonly used, nonspecific kinase substrates such as bovine MBP 52 get relatively high MIscore values due to their widespread use in in vitro kinase assays. These molecules obviously do not represent specific, physiological substrates of LRRK2 but achieve a high MIscore due to their repeated use. Conversely, a low MIscore value may not mean that the interaction is a false positive, but rather that it is currently supported by limited evidence. Combining our “close-to-native” network information with MIscore confidence cutoffs yields a very small number of potential bona fide LRRK2 interacting partners. Three of the interacting pairs, LRRK2-SNCA, LRRK2-RPS15, and LRRK2-LAMP2, have only been detected in native conditions as colocalizations, which is not definitive evidence for a physical interaction, and orthogonally confirmed in coimmunoprecipitation experiments in human cell lines 53–56. When using a high-confidence cutoff (MIscore ≥ 0.60) only the LRRK2 self-interaction and LRRK2-RPS15 and LRRK2-PRDX3 interactions remain among those detected under “close-to-native” conditions. The very low number of putative interacting partners tested under these conditions suggests the technical limitations of approaches using native LRRK2.

LRRK2 self-interaction is one of the best-documented interactions in IntAct, being represented by a total of 83 experimental pieces of evidence from 28 different publications detected by mainly in vitro kinase assays, plus coimmunoprecipitation, cosedimentation, yeast two-hybrid assays, protein arrays, pull-downs, and electron microscopy. The overabundance of autophosphorylation data is due to the interest in the G2019S PD-causing mutation in the LRRK2 kinase domain. Although quite some effort has been spent on the identification of LRRK2 kinase substrates and various candidates arose from in vitro studies (summarized in Fig. 7), no direct phosphorylation of these bona fide substrates by LRRK2 has been unambiguously demonstrated in mammalian cells 57.

If we combine our three selection criteria, there are only two proteins detected among human LRRK2 interactions with a high MIscore, in native conditions and phosphorylated by LRRK2 in vitro: RPS15 and PRDX3. The LRRK2-RPS15 interaction has recently been found via coimmunoprecipitation in human cell lines and via confocal microscopy in human neurons and RPS15 has been identified as substrate in in vitro kinase assays 55,56. The LRRK2-PRDX3 interaction is detected in in vitro kinase assays as well as with confocal microscopy in *Drosophila melanogaster* brain expressing human proteins, yeast two-hybrid, and coimmunoprecipitation in human cell lines 58,59. RPS15 is a constitutive ribosomal protein and it has been shown that LRRK2 can have an influence in translation initiation modulation through phosphorylation of RPS15 56. PRDX3 is a mitochondrial antioxidant enzyme involved in maintaining redox homeostasis and this interaction could further link mitochondrial dysfunction and PD 60. While the characterization of these as potential LRRK2 substrates based purely on interaction data is currently more convincing than other human LRRK2 interacting partners, it is important to note that all the interactions reported for each come from only two different publications and in the case of PRDX3 both come from the same group. Further validation by different laboratories is required before identifying them as bona fide LRRK2 substrates.

## 4. Reactome pathway annotation analysis

**Figure 8:**
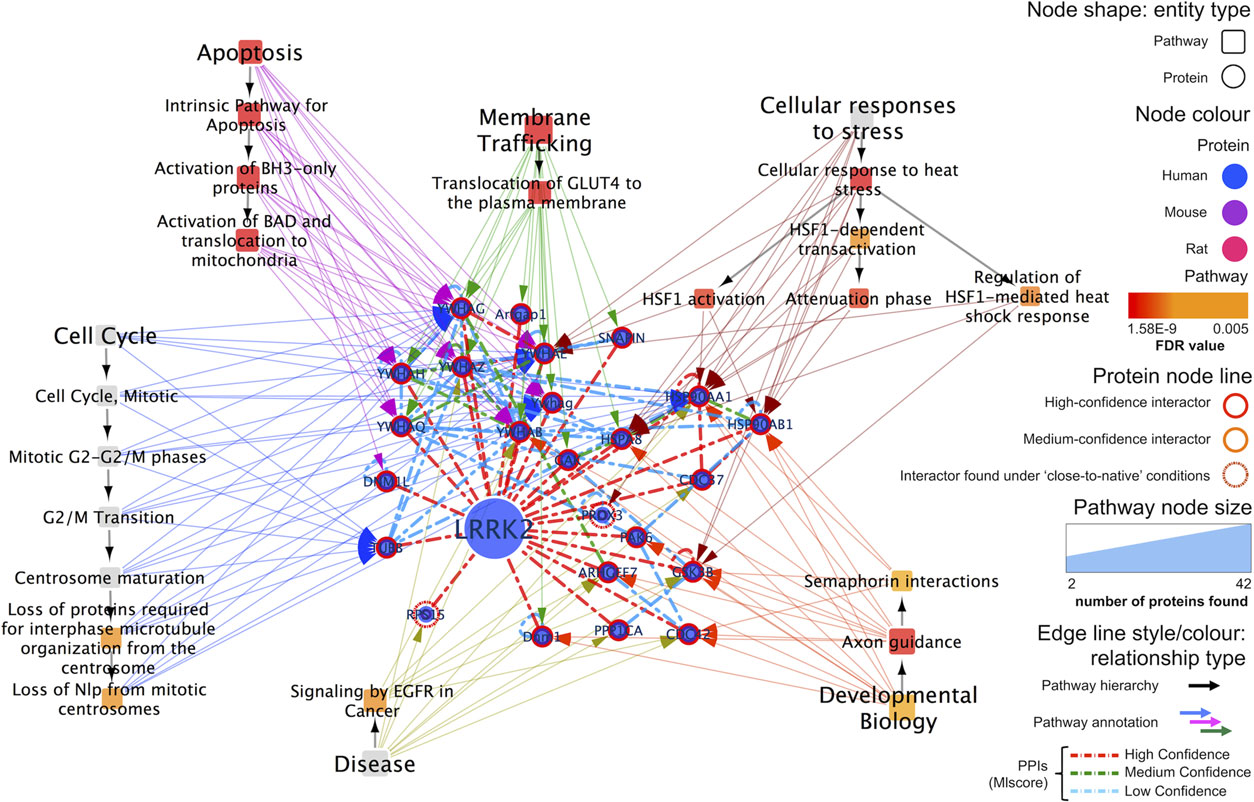
LRRK2 high-confidence interacting partners analyzed using the Reactome analysis tool. LRRK2 high-confidence partners and the interactions between them are represented associated to Reactome pathways that appear to be significantly enriched for this group of proteins. Each pathway is associated with annotated proteins through colored arrows and Reactome's pathway hierarchy is shown using black arrows. See legend for the remaining visual features used in this representation. A web visualization of this network can be found at http://tinyurl.com/lrrk2-f8.

In order to obtain a more global perspective on the putative interacting partners of LRRK2, we used the pathway-enrichment analysis tool offered by the annotated pathway database Reactome 61 to highlight pathways in which human LRRK2 interacting partners are represented (Fig. 8). Term-enrichment analysis 40 uses a hypergeometric-type statistical test to identify which annotations to a term (Gene Ontology annotation or KEGG 62 or Reactome pathway name) are significantly overrepresented in the complete set of annotations for a protein list. Although this type of analysis is most effective with a large number of proteins/genes, visualizing this kind of information in combination with physical interaction information can help identify potential functional modules. However, building such a representation remains a challenge. There are several tools that can help with computationally generated visualizations such as the Cytoscape apps BiNGO 63 and ClueGO 64, web-based integration suite GeneMANIA 65, or commercial analysis suite Ingenuity Pathway Analysis (IPA, www.ingenuity.com), and the decision on which one to use often needs to be made on a case-by-case basis.

We have used the Reactome pathway analysis tool (www.reactome.org/PathwayBrowser/#TOOL=AT) for annotation enrichment using lists of LRRK2 interacting proteins (Supporting Information Tables 4 and 5) and we represented the results using Cytoscape again. The Reactome pathway analysis tool performs a binomial test implemented in the COLT library (http://acs.lbl.gov/ACSSoftware/colt) to determine which pathways are overrepresented in the list of proteins/genes in comparison to the full Reactome database (http://wiki.reactome.org/index.php/Usersguide#Pathway_Analysis). Since Reactome is human-centric, annotations to mouse proteins/genes are inferred by ortholog mappings derived from Ensembl Compara (www.ensembl.org/info/genome/compara). We performed the analysis using high- and medium-confidence interacting partners of human LRRK2. LRRK2 itself, small molecules, and proteins used exclusively in in vitro kinase assays (e.g. as nonphysiological substrates) were excluded from the analysis. For ease of visualization, only the results for the top terms (false discovery rate < 0.005) were represented, the full result of the analysis for the high-confidence interacting partners can be found in Supporting Information Table 4. The result file was then transformed so each line represents a pathway identifier paired with a protein annotated to it and this was imported as a network into Cytoscape. This network was merged with protein interactions between LRRK2 interactors obtained using the built-in PSICQUIC client of Cytoscape and coming only from IMEx-compliant databases, along with a network of hierarchical pathway relationships (http://www.reactome.org/download/current/ReactomePathwaysRelation.txt). The analysis was represented as a network in Fig. 8 and Supporting Information Fig. 4.

Figure 8 shows a summarized version of the analysis performed for high-confidence interacting partners of human LRRK2. An analysis including medium-confidence interacting partners of LRRK2 can be seen in Supporting Information Fig. 4 and Table 5. Due to its complexity, the Reactome analysis networks (Fig. 8 and Supporting Information Fig. 4) have been represented in a separate Cytoscape session file available as Supporting Information(lrrk2_reactome_analysis.cys).

We have chosen to represent the most significant pathways connected to LRRK2 high-confidence interactors by arrows and to overlay the interactions between the proteins to group them in clusters when possible. The pathways are arranged using the hierarchy of the Reactome database, where each general, high-level pathway is divided into a number of subpathways that represent more-specific processes. Each subpathway node is related to the proteins within it, providing the user with a map in which proteins can be grouped into functional subclusters and giving a visual overview of the current knowledge about LRRK2 interacting partners.

The analysis illustrated in Fig. 8 shows the main pathways in which LRRK2 interacting partners are involved and the physical interactions between these molecules. Regulation of cell cycle (possibly through centrosome maturation), the intrinsic pathway of apoptosis, axon development (controlled through semaphorin interaction), and membrane trafficking, along with response to stress and signaling by EGFR, are found to be linked to high-confidence LRRK2 interacting partners. Regulation of membrane trafficking and apoptosis are linked to a cluster of interactions involving 14-3-3 regulatory proteins and SNAPIN 66, a protein involved in vesicular trafficking in the synapse 67,68, a role that has also been proposed for LRRK2 36,37,69,70. Most of the proteins in the cluster are connected through low-confidence interactions, so care must be taken when interpreting these results, especially since 14-3-3 proteins share great sequence homology and a tendency to dimerize 71, which can result in a number of artifactual heterodimers being reported. Nevertheless, we can detect the high-confidence interaction between YWHAE and YWHAZ, a well-characterized heterodimer. This family of proteins is known for regulating a broad variety of cellular processes by binding to different signaling proteins, such as kinases or phosphatases mediated by specific motifs containing phosphorylated residues 72. Different phosphorylated LRRK2 residues have been described as 14-3-3 binding sites, including phospho S910, S935, and S1444 73,74. 14-3-3 binding is reduced upon LRRK2 kinase inhibition 74, which blocks LRRK2 secretion in exosomes 75. HSP90-alpha/beta (HSP90AA1/HSP90AB1) and its co-chaperone CDC37, known to mediate the binding of HSP90 to kinases 76, form another small cluster of interacting proteins 26, associated to cell cycle control, response to stress, and axon development. Other proteins such as GSK3B 77, PAK6 38,55, CDC42 78,79, and ARHGEF7 79 (two kinases, a GTPase, and a guanine nucleotide exchange factor, respectively) form a loosely connected cluster that seems related to axon development.

Additional insight may be gained by using medium-confidence interacting partners of LRRK2 to identify additional subpathways from Reactome (Supporting Information Fig. 4). Several specific signaling pathways are highlighted including WNT (linked to LRRK2 in 80), NOTCH, NGF, and Hedgehog pathways. Relationships between control of the cell cycle and centrosome maturation in the transition between G2 and M phases are revealed and also a previously described link between LRRK2 and innate immune function 81 has been confirmed. This analysis brings out a link to regulatory RNA pathways previously overlooked. A role in microtubule dynamics and cytoskeleton organization has been proposed for LRRK2, seemingly shared by another PD-related protein, synuclein 82,83. Several tubulins and synuclein can be found as medium-confidence LRRK2 interactors in our dataset. Another important neurodegeneration-related protein, tau (MAPT), is found among the high-confidence interacting partners of LRRK2 and has been linked to microtubule stability 84,85. Synuclein has been found to colocalize with LRRK2 in human brain tissue and the proteins co-immunoprecipitate in cell culture, but it has yet to be identified as a LRRK2 substrate 53. Different forms of MAPT/tau (Tau-B, Tau-E, Tau-F, and fetal Tau) have been identified in vitro as kinase substrates for LRRK2 (see Fig. 4) and their interaction confirmed through in vitro pull-down assays and coimmunoprecipitations in SH-SY5Y cell lines 84,86, so this is a more probable LRRK2 substrate. Other PD-related proteins, such as PARK2, PINK1, or DJ1, have been linked to LRRK2 through genetic interaction studies 87, but only low-confidence physical evidence for PARK2 as an interacting partner has been found. The results of this analysis, limited as it is and exclusively focused around LRRK2 immediate interacting partners, still managed to provide a representative picture of the current knowledge surrounding this protein.

## 5. Concluding remarks

In this review we show how the rich detail in which interaction experiments are represented using the IMEx curation guidelines allows for advanced visualization options for the interactome. Using LRRK2 as an example, we have illustrated how targeted curation, followed by an accurate visualization of data and its integration with other resources, can help in providing a comprehensive vision of the biology of a given domain.

Focusing exclusively on the interaction data, we have illustrated how different aspects of the information underlying the interactome can be brought to light with an effective visualization, helping the researchers to focus in specific areas while hopefully filtering out part of the noise inherent to PPI information. Visualizing current interactome knowledge in a network brings together data from large-scale, relatively unbiased datasets with the topic-focused, small-scale publications that are recorded via targeted curation. We have created networks in which the methodology for the interaction detection, host system and nature of the protein constructs, and finally an experimentally derived confidence score (MIscore) have been used to highlight certain LRRK2 interaction candidates above others. While MIscore can be calculated with data from any database that provides a minimum of information as specified in the MIMIx standard 17, it is important to stress that identifying “close-to-native” conditions and the identification of LRRK2 role as enzyme or substrate in the enzymatic assays are only possible when using highly detailed data presented in the PSI-MITAB 2.7 or PSI-XML 2.5 formats.

Our visual review of the LRRK2 interactome shows how, despite large amounts of interaction evidence, lack of orthogonal cross-validation makes the identification of bona fide, biologically meaningful LRRK2 interacting partners a challenging task. Nevertheless, promising candidates can be readily identified from the information stored in IntAct and other databases. LRRK2-related interactions are still being actively curated by the IntAct team, and this hopefully will increase the number of validated LRRK2 interacting partners in the future.

Despite its limitations, data visualization and network/pathway analysis may increasingly improve our understanding of basic biology and suggest new therapeutic targets in poorly characterized diseases. In addition, a protein-interaction network may link several disease-associated proteins allowing the identification of converging pathomechanisms and, following the diseasome theory 88, to propose novel disease proteins. Targeted, in-depth curation approaches greatly improve the representation of specific domains of knowledge in public repositories while an effective visualization strategy enriches and improves the analyses and strengthens the conclusions that can be drawn from them.

## Abbreviations

APID: Agile Protein Interaction DataAnalyzer
DIP: Database of Interacting Proteins
FDR: False Discovery Rate
HIPPIE: Human Integrated Protein-Protein Interaction rEference
HPRD: Human Protein Reference Database
IMEx: International Molecular Exchange consortium
IPA: Ingenuity Pathway Analysis
LEAPS: Linked Efforts to Accelerate Parkinson's Solutions
MINT: Molecular INTeraction database
MIscore: Molecular Interactions score
OMIM: Online Mendelian Inheritance in Man
PD: Parkinson's disease
PPI: protein–protein interaction
PSI: Proteomics Standards Initiative
PSICQUIC: Proteomics Standard Initiative Common Query Interface
PSI-MI: Proteomics Standards Initiative for Molecular Interactions
STRING: Search Tool for the Retrieval of Interacting Genes/Proteins
UniHI: Unified Human Interactome

## Supporting Information

As a service to our authors and readers, this journal provides supporting information supplied by the authors. Such materials are peer reviewed and may be re-organized for online delivery, but are not copy-edited or typeset. Technical support issues arising from supporting information (other than missing files) should be addressed to the authors.

Table S1. Table S2. Table S3. Table S4. Table S5. Figure S1. Figure S2. Figure S3. Figure S4.
