# Supplementary material for: A visual review of the interactome of LRRK2: Using deep-curated molecular interaction data to represent biology
Source: Proteomics. 2015 Mar 21;15(8):1390–404. doi: 10.1002/pmic.201400390 (PMC4415485; doi:10.1002/pmic.201400390)
Supplement: Supplementary file 1 — Table S1. Table S2. Table S3. Table S4. Table S5. Figure S1. Figure S2. Figure S3. Figure S4. [file pmic0015-1390-sd1.zip › pmic201400390-sup-0006-Supplementary figure legends_revised_2.docx]

**Supplementary figure legends**

Supplementary figure 1 (related to figure 2A): Complete list of detection methods listed as evidence for LRRK2/Lrrk2 interactions. PSI-MI controlled vocabulary terms are used to define the methods, and the number of evidences found for each method is indicated by its name.

Supplementary figure 2 (related to figure 3): LRRK2/Lrrk2 interaction network with edge color mapped to host organism. A web visualization of this network is available at <http://tinyurl.com/lrrk2-fS2>.

Supplementary figure 3 (related to figure 7): LRRK2/Lrrk2 interactions derived from *in vitro* enzymatic assays. Enzymatic assay type is represented as the color of the edge, edge arrow style indicate specific biological roles for the participants in the interaction, for example enzyme-substrate relationships or inhibitor roles. A web visualization of this network is available at <http://tinyurl.com/lrrk2-fS3>.

Supplementary figure 4 (related to figure 8): LRRK2 medium-confidence interacting partners analyzed using the Reactome analysis tool. See figure 4 for network legend. High- and medium-confidence LRRK2 interactors can be distinguished by node border color (high = red, medium = orange). A web visualization of this network is available at <http://tinyurl.com/lrrk2-fS4>.
