# Supplementary figures and images for: A visual review of the interactome of LRRK2: Using deep-curated molecular interaction data to represent biology
Source: Proteomics. 2015 Mar 21;15(8):1390–404. doi: 10.1002/pmic.201400390 (PMC4415485; doi:10.1002/pmic.201400390)

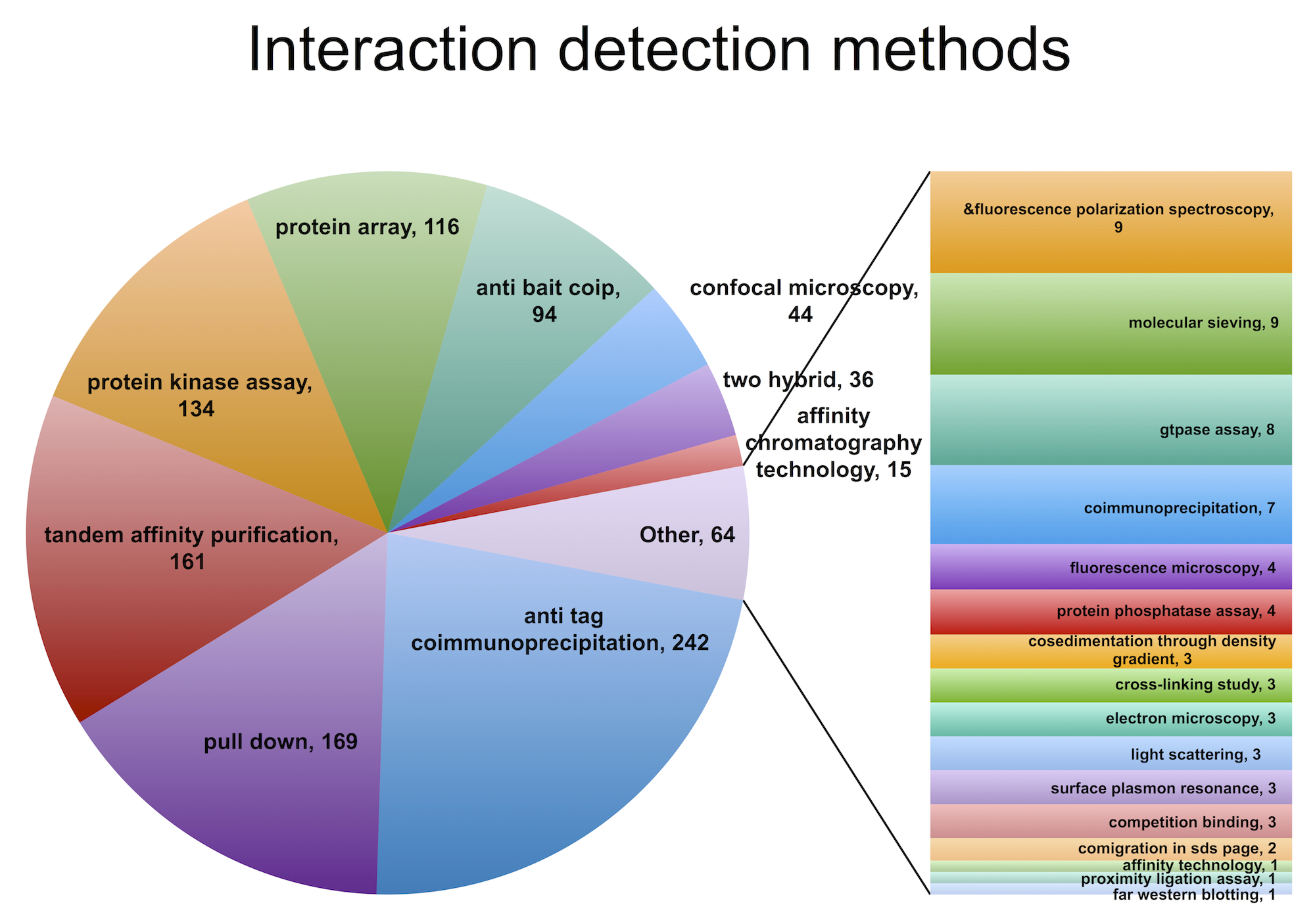

Supplement: Supplementary file 1 — Table S1. Table S2. Table S3. Table S4. Table S5. Figure S1. Figure S2. Figure S3. Figure S4. [file pmic0015-1390-sd1.zip › pmic201400390-sup-0001-supplfig1.jpeg]

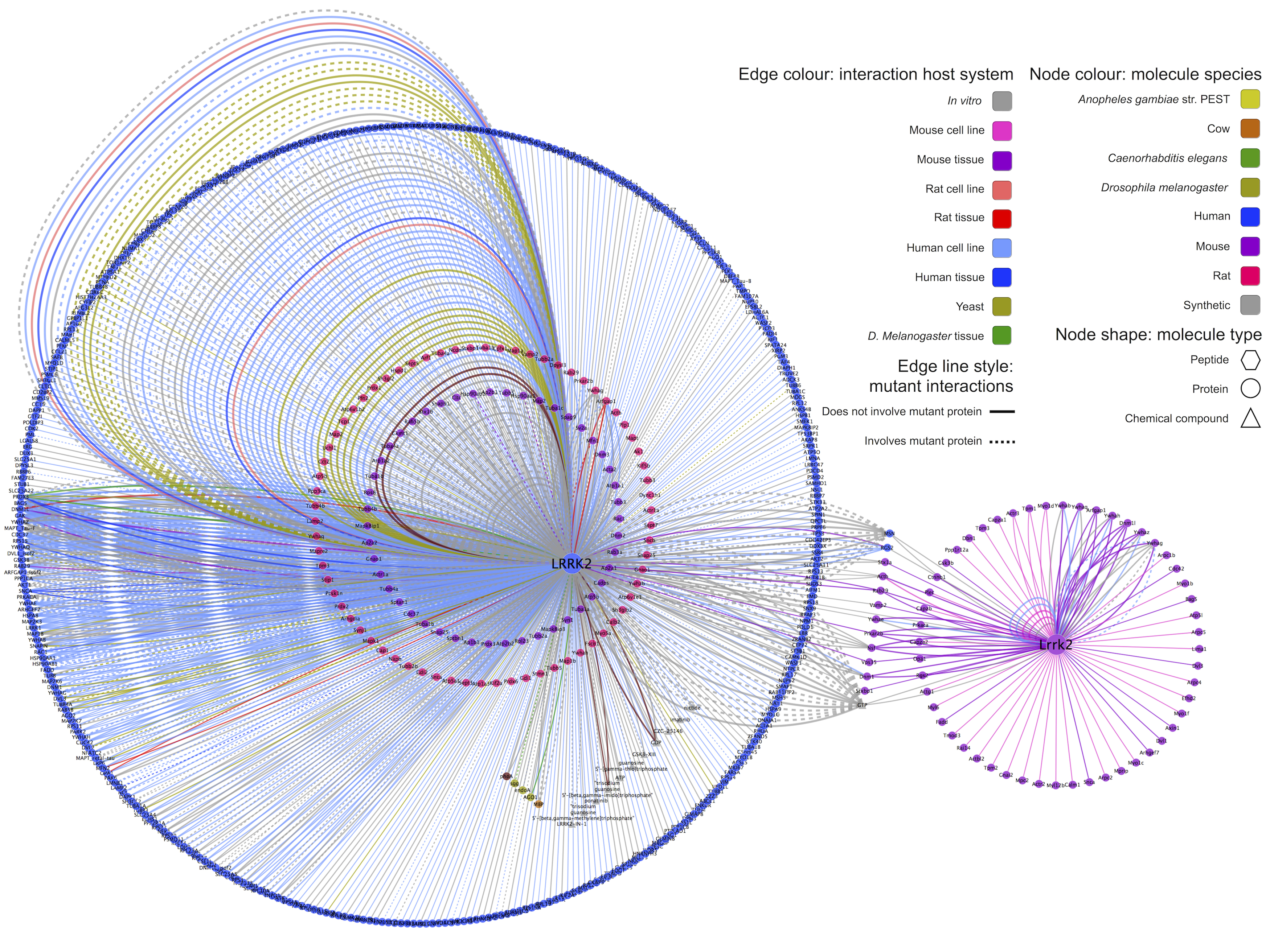

Supplement: Supplementary file 1 — Table S1. Table S2. Table S3. Table S4. Table S5. Figure S1. Figure S2. Figure S3. Figure S4. [file pmic0015-1390-sd1.zip › pmic201400390-sup-0002-supplfig2.jpeg]

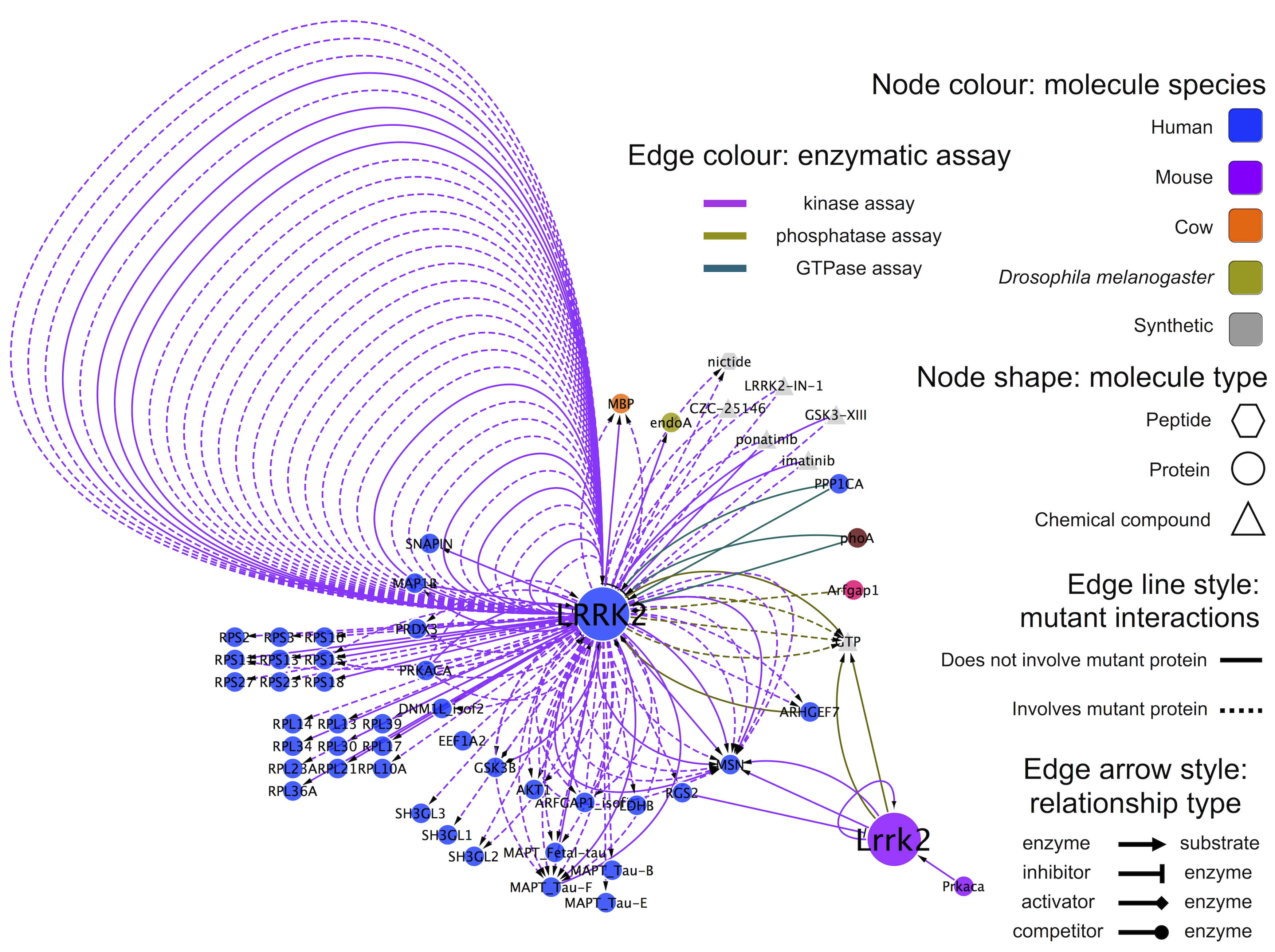

Supplement: Supplementary file 1 — Table S1. Table S2. Table S3. Table S4. Table S5. Figure S1. Figure S2. Figure S3. Figure S4. [file pmic0015-1390-sd1.zip › pmic201400390-sup-0003-supplfig3.jpeg]

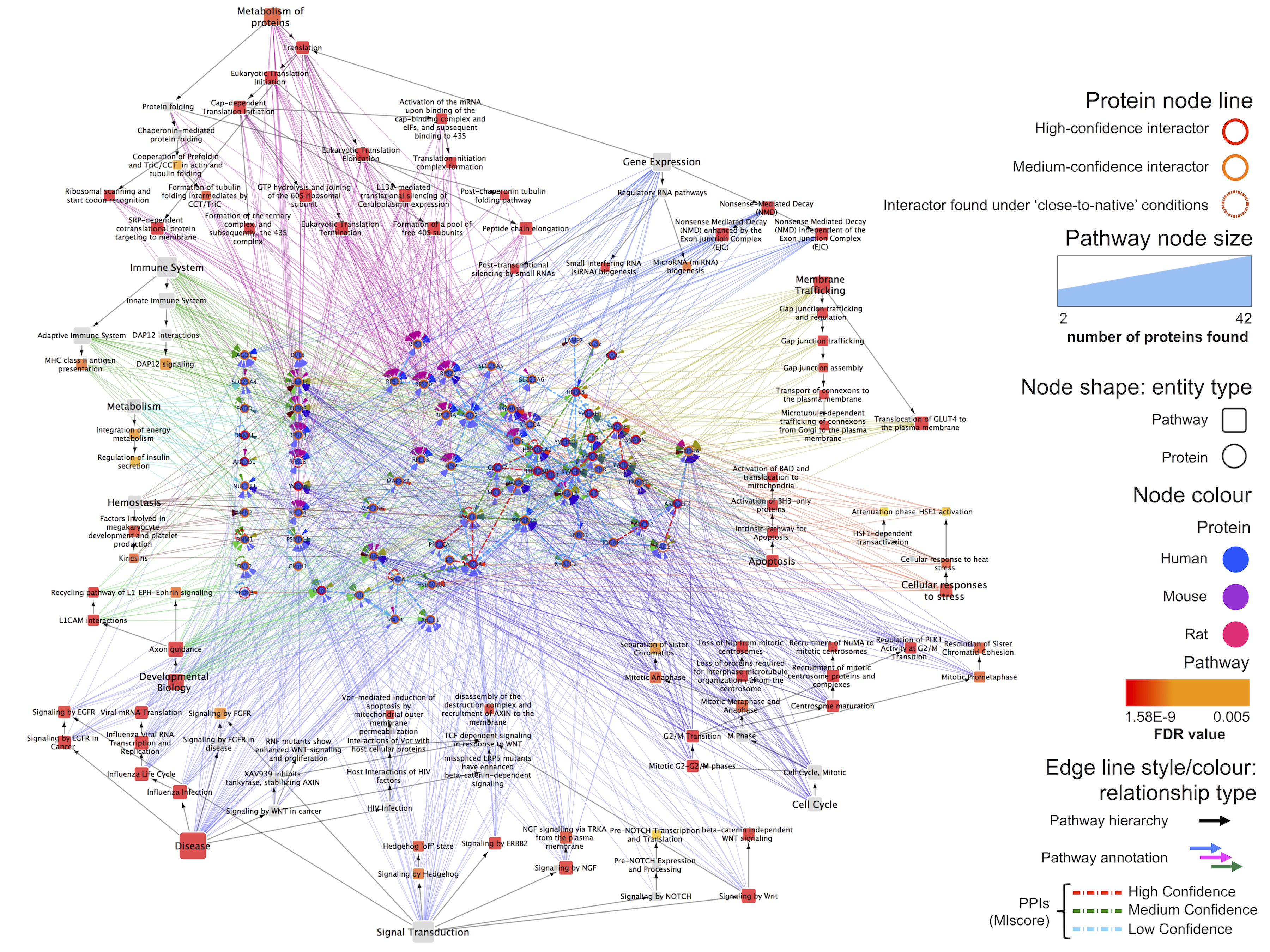

Supplement: Supplementary file 1 — Table S1. Table S2. Table S3. Table S4. Table S5. Figure S1. Figure S2. Figure S3. Figure S4. [file pmic0015-1390-sd1.zip › pmic201400390-sup-0004-supplfig4.jpeg]
